# Diagnostic Accuracy of Utilizing Artificial Intelligence for Malaria Diagnostic: A Systematic Review and Meta-Analysis

**DOI:** 10.3390/idr18010011

**Published:** 2026-01-13

**Authors:** Icha Farihah Deniyati Faratisha, Khadijah Cahya Yunita, Hanifa Rizky Rahmawati, Loeki Enggar Fitri, Nuning Winaris, Lailil Muflikah

**Affiliations:** 1 Master Program in Biomedical Science, Faculty of Medicine, Universitas Brawijaya, Malang 65145, Indonesia; 2 ATOM Research Group, Faculty of Medicine, Universitas Brawijaya, Malang 65145, Indonesia; khadijah.cahya@gmail.com (K.C.Y.); rizkyhanifa19@student.ub.ac.id (H.R.R.); nuning_winaris@ub.ac.id (N.W.); 3 Department of Clinical Parasitology, Faculty of Medicine, Universitas Brawijaya, Malang 65145, Indonesia; 4 Department of Informatics Engineering, Faculty of Computer Science, Universitas Brawijaya, Malang 65145, Indonesia; lailil@ub.ac.id

**Keywords:** artificial intelligence, diagnostic accuracy, malaria, microscopy, polymerase chain reaction

## Abstract

Background: Malaria remains a major public health concern around the world. Microscopic blood smear examination continues to be the gold standard for diagnosis; however, it requires high technical skills and expertise, limiting diagnostic accuracy in resource-poor settings. Artificial intelligence (AI) has emerged as a promising tool to support malaria detection. This systematic review provides an overview of the diagnostic performance of AI-based systems for malaria diagnosis in a clinical setting. Methods: This study followed the Preferred Reporting Items for Systematic reviews and Meta-Analyses (PRISMA) guidelines and involved articles within the last 10 years that were collected from PubMed, ScienceDirect, Cochrane, EBSCO, and Wiley Online Library. Original articles that reported AI diagnostic accuracy with external validation were involved. The quality of each study was evaluated using the Quality Assessment of Diagnostic Accuracy Studies (QUADAS-2). Results: Ten studies with 6754 patients were analyzed. Pooled results of sensitivity [87.7% (95% CI: 78.2–93.4)] and specificity [91.4% (95% CI: 77.3–97.1)] revealed how much the AI agrees with each method when that method is used as a gold standard. Additionally, AI achieved a sensitivity of 87.7% and a specificity of 91.4% compared to microscopy examination and a sensitivity of 90.7% and a specificity of 88.3% compared to polymerase chain reaction (PCR). Conclusions: AI-based systems improve malaria diagnosis by providing high accuracy, automation, and lower costs. Showing performance comparable to reference methods such as microscopy and PCR, AI is a promising complementary tool for malaria control.

## 1. Introduction

Malaria is a life-threatening disease transmitted by female *Anopheles* mosquitoes and caused by *Plasmodium* spp. [1]. Globally, Malaria is predicted to affect 263 million people worldwide, with 597,000 deaths occurring in 83 countries [2]. The prevalence of malaria cases in Indonesia is estimated at 1.1 million cases with 1900 deaths [3]. Along with India, Indonesia ranks among the highest malaria prevalence and mortality rates in Southeast Asia and South Asia [2]. Although currently, 77% of regions in Indonesia are declared malaria-free, malaria cases remain very high in Eastern Indonesia, especially Papua Province [4].

One of the challenges in eliminating malaria is the emergence of resistance to antimalarial medications and insecticides [5]. Antimalarial drug resistance reduces the success of therapy and increases the risk of complications and transmission, while insecticide resistance reduces the effectiveness of vector control [6]. Globally, there have been reports of resistance to commonly used antimalarial drugs, and resistance has emerged to almost all categories of antimalarial drugs. Now, the current recommended treatment is artemisinin combination therapy (ACT), which involves the use of two active pharmaceuticals with distinct mechanisms of action: an artemisinin derivative and a partner drug [7]. Moreover, despite the fact that malaria vaccines have been a significant area of research for numerous years, it was only in 2021 that the RTS,S/AS01 vaccine gained endorsement from the World Health Organization (WHO), followed by the R21/Matrix-M vaccine in 2023 [8]. Both vaccines have been advised for the prevention of *Plasmodium falciparum* malaria infection, especially among children residing in endemic regions, with an emphasis on moderate to high transmission [6].

Accurate malaria diagnosis and timely therapy are prerequisites for effective malaria management. In areas with high malaria incidence, the process of diagnosing and detecting malaria through blood smear examination is a complex laboratory task and increases the workload for clinical parasitologists [4]. The use of microscopy as a method for examining blood smears remains a gold standard for diagnosis globally. This is because the method is considered inexpensive, rapid, and universal. However, microscopic examination requires a high level of skill and experience to ensure accurate diagnosis, making this method a major challenge in malaria control efforts [9]. In some recent studies, microscopists detected fewer malaria cases than polymerase chain reaction (PCR), with a sensitivity ranging from 73 to 88%, with false-positive rates up to 47% [10,11,12]. Additionally, the deletion of pfhrp2 and/or pfhrp3 genes leads to false negatives in malaria rapid diagnostic tests (RDTs). The deletion of pfhrp2/3 genes complicates malaria detection has been observed in 42 countries, which poses a risk to malaria control strategies [6,13].

The challenges of effective malaria diagnosis have led to the development of artificial intelligence (AI) systems for detecting *Plasmodium* spp. [9,14]. AI can assist in identifying infected and uninfected erythrocytes using deep learning-based models [15]. Various deep learning methods, including neural networks, convolutional neural networks (CNNs), and convolutional encoder–decoders, have been developed, focusing on three main areas: image segmentation, image quality enhancement, and particle tracking [16]. One example is the iMAGING AI-based Diagnostic System, a fully automated malaria diagnostic platform that integrates AI-driven image analysis with a robotized microscope. This system analyzes Giemsa-stained blood smears using CNNs trained on labeled malaria datasets to determine the presence of *Plasmodium* parasites. The microscope and AI model are operated via a smartphone or computer application, enabling automated diagnosis with minimal operator intervention [15].

Similarly, Liu et al. developed an artificial intelligence-based object detection system for malaria diagnosis (AIDMAN). This system integrates the YOLOv5 model with the Transformer model to execute the complete process, ranging from image analysis to the diagnosis of malaria [9]. These AI-driven image analysis techniques allow for reliable detection of *Plasmodium* spp. in digital images by training pre-trained models on large datasets, thereby emulating conventional optical microscopy of thin and thick blood smears while automating the examination process [10].

While microscopic examination remains rapid and inexpensive, AI is considered a potential solution to existing testing limitations by providing more reliable diagnoses, reduced screening costs, improved healthcare access, and reduced physician workload. AI-driven tools can assist in managing diagnostic workflows by automating routine tasks with large datasets quickly, allowing physicians to focus on more complex cases, thereby improving efficiency in parasite detection [17]. Furthermore, the use of smartphones that can be integrated with AI-based image analysis technology offers a valuable option, especially in resource-limited settings in endemic areas [15].

Although various artificial intelligence models have been developed and trained to detect malaria parasites, their application in clinical practice still faces several challenges. These include limited datasets, a limited number of datasets with low variation in red blood cells and parasite morphology, and the use of data generally sourced from the National Institutes of Health (NIH), which often exhibits overlapping between cells and overly idealized staining results. Furthermore, most AI-based studies for malaria detection still focus on internal validation, thus limiting the applicability and generalizability of their results to clinical setting conditions [9]. Therefore, this systematic review provides an overview of the diagnostic performance of AI for malaria detection, focusing on studies that use robust reference standards and external validation in clinical settings.

## 2. Materials and Methods

### 2.1. Data Sources and Search Strategy

We conducted a systematic review using the Preferred Reporting Items for Systematic Reviews and Meta-Analyses (PRISMA) 2020. The literature review covered data sources such as PubMed, ScienceDirect, Cochrane, EBSCO, and Wiley Online Library over the past 10 years. Boolean operators (OR and AND) were used to obtain the desired results. The search terms were (“Artificial Intelligence” OR “Machine Learning” OR AI OR “Machine intelligence” OR CNN OR “Deep Learning”) AND (“Malaria” OR “Infection, Malaria” OR “*Plasmodium* infection”) AND (“Diagnosis” OR “Diagnostic” OR “sensitivity” OR “specificity”).

### 2.2. Study Selection

Three authors independently identified reliable studies from each data source. All retrieved records were imported into Mendeley reference management software (version 2.82.0, Elsevier, Amsterdam, The Netherlands) and the duplications were identified using a manual screening process by comparing titles, authors, publication years, journal names, and digital object identifiers (DOI).

We reviewed the full text of potential scientific articles and assessed their compliance with the established inclusion criteria. Eligibility criteria included: study design in the form of an original article accessible in full (full text), discussion of malaria diagnostic methods using artificial intelligence (AI, deep learning, machine learning, convolutional neural networks, etc.), study outcomes in the form of AI method accuracy (sensitivity, specificity, area under the curve), and studies conducted only in human studies with clinical validation.

Studies were excluded if the article was published before 2015, was a pre-print article, was a chapter, proceeding paper, guideline, review, or commentary, contained duplicate publications, had incomplete data, included non-peer-reviewed articles, or was written in a language other than English. and was written in a language other than English.

### 2.3. Data Extraction

The authors independently extracted relevant data using a standardized form, including the first author and year of study, location/setting, number of patients, gold standard examination (polymerase chain reaction/expert microscopy), AI index, type of validity, units used, true positive (TP), false positive (FP), true negative (TN), false negative (FN) rates, and other annotations.

### 2.4. Study Outcomes

The primary outcome of this study included the overall pooled sensitivity and specificity of the diagnostic method using artificial intelligence. We also analyzed subgroups, such as comparing AI with polymerase chain reaction (PCR) and AI with expert microscopy.

### 2.5. Statistical Analysis

MetaDTA: Diagnostic Test Accuracy Meta-Analysis v2.01 Shiny App (NIHR Complex Reviews Support Unit, University of Leicester, Leicester, UK) [18] was used to generate forest plots, summary receiver operating characteristic (SROC) plots, and summary sensitivity and specificity plots using a bivariate random effects model.

### 2.6. Quality Assessment

Study quality was evaluated using the Quality Assessment of Diagnostic Accuracy Studies (QUADAS-2) instrument, a validated methodological instrument developed by Whiting et al. (University of Bristol, UK) [19] for patient selection, performance index tests, performance reference tests, as well as time and flow.

## 3. Results

### 3.1. Selection Findings

**Figure 1:**
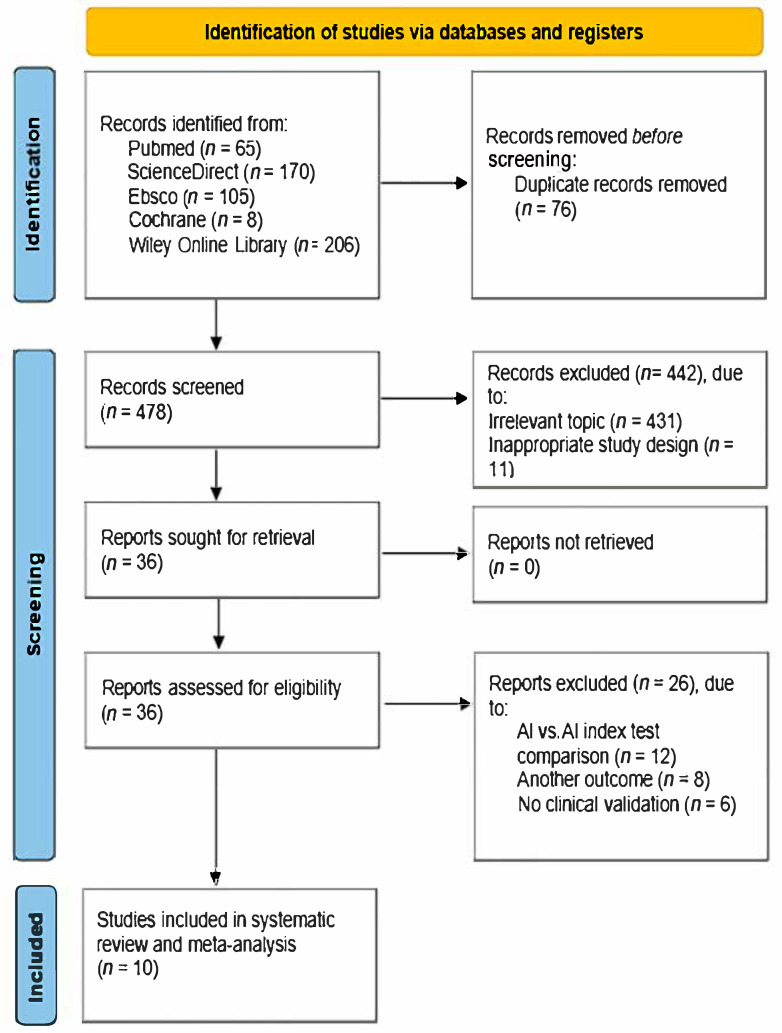
Flowchart of the study selection process using PRISMA.

We identified 554 articles related to artificial intelligence and malaria diagnosis through online scientific databases according to the search descriptions of the databases. After excluding duplicate articles, we found 478 articles. We screened for unrelated articles (*n* = 431) and inappropriate study designs (*n* = 11). In addition, exclusions were made by determining inappropriate eligibility criteria, such as comparisons only with individual AI model indices (*n* = 12), inappropriate study outcomes (*n* = 8), and lack of clinical validation (*n* = 6). Ultimately, 10 studies were included in this study. Figure 1 illustrates the study selection process using PRISMA.

### 3.2. Study Characteristics

This study included 6754 patients from various countries, including those with malaria endemic areas, a history of travel to endemic areas, or exposure from relatives who traveled from endemic areas, such as Spain, Sierra Leone, the United States, Ghana, Ethiopia, the United Kingdom, Burkina Faso, Kenya, the Republic of Congo, Senegal, South Africa, Uganda, Bangladesh, Cambodia, Nepal, Thailand, Brazil, Sudan, and Peru. Table 1 describes the study characteristics of each included study. Detailed raw data related to sampling sites, examined species, and comparative diagnostic techniques from each study can be found in the Supplementary Materials File S1.

### 3.3. Risk of Bias Assessment

**Figure 2:**
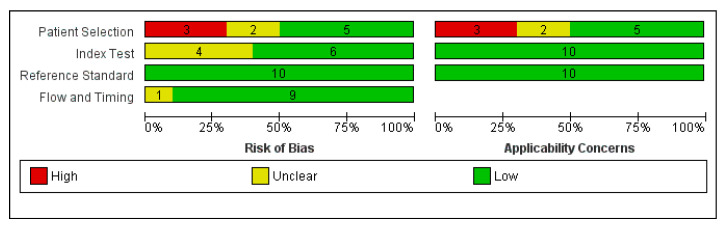
Risk of bias assessment using QUADS-2.

Risk of bias was assessed using the QUADAS-2 instrument. Figure 2 depicts the QUADAS-2 chart of the ten reviewed studies. Most of these studies were of good methodological quality, although some weaknesses persisted.

Of the 10 studies included in this meta-analysis, 3 of the 10 (33%) demonstrated a risk of bias in patient selection, with some using retrospective data, dataset-based studies, or special populations (e.g., travelers/migrants). Two other studies did not detail their patient recruitment methods and were therefore deemed unclear (20%). Regarding the index test, 4 of the 10 studies (40%) did not explicitly report blinding and were therefore categorized as unclear, but none of the studies were considered high risk. The reference standard consistently had a low risk because almost all used trained microscopists and/or PCR testing, the gold standard for malaria diagnosis. The flow and timing aspects were also mostly assessed as good, as the index test and reference standard were performed on the same specimens at short intervals, although one study was considered unclear (10%) due to its dataset-based nature.

In the applicability domain, 5 of the 10 studies had a high or unclear risk of applicability (50%), particularly in the patient selection domain, with 3 of the 10 (30%) categorized as high risk and 2 of the 10 (20%) categorized as unclear. This is because the studies were conducted on travelers in non-endemic countries or were dataset-based, with low applicability to populations in endemic areas. For the other domains, the index test, reference standard, and flow and timing generally did not raise any applicability concerns.

Overall, the assessment results show that the main strengths of the studies were the strong reference standard and the appropriateness of the examination timeline, while the main weaknesses were patient selection and the reporting of blinding of the index test.

### 3.4. Study Outcomes

Ten studies were identified that met the eligibility criteria for this systematic review and meta-analysis. Sample sizes varied across the included studies, ranging from 46 patients to 2250. The types of AI used varied. Most studies (80%) utilized a Convolutional Neural Network (CNN) approach, either in the form of EasyScan GO [21,22,23], Autoscope [26], or Malaria Screener and PVF-Net [24]. Additionally, YOLOv5-based CNN models were also used, such as in AIDMAN [9] and iMAGING [15]. Several other studies (20%) evaluated the MiLab deep learning platform [14,20,25], both in laboratory and field settings. The reference standards used varied widely, ranging from expert microscopy, PCR/qPCR, to RT-PCR. Five of the ten studies (50%) combined more than one reference standard, with PCR as molecular confirmation.

**Figure 3:**
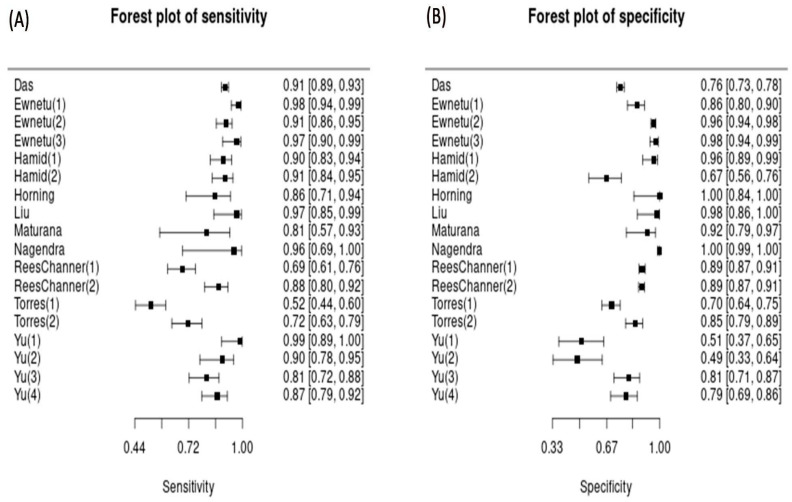
Forest plot for performance across studies included in this meta-analysis. The pooled sensitivity (**A**) and specificity (**B**) were conducted in studies of all diagnostic methods (PCR and microscopy) with 95% confidence intervals. Generated by MetaDTA: Diagnostic Test Accuracy Meta-Analysis v2.01 Shiny App. Each study labeled (1), (2), (3), and so forth denotes variations in sampling sites, examined species, and comparative diagnostic techniques. Detailed raw data can be found in the Supplementary Materials File S1.

**Figure 4:**
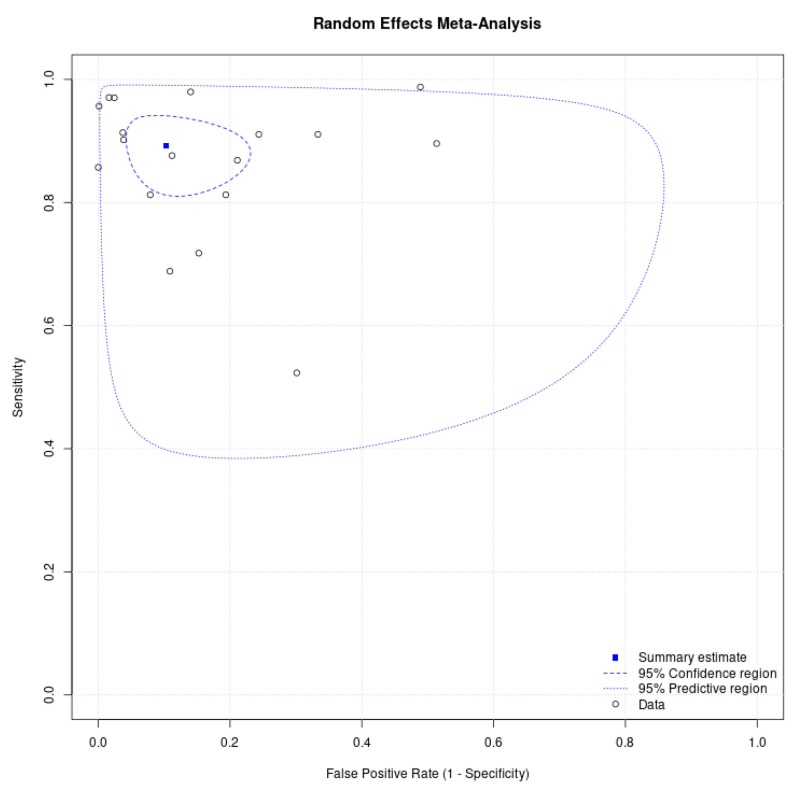
Summary receiver operating characteristic (ROC) of AI application for malaria diagnosis generated from MetaDTA: diagnostic test accuracy meta-analysis v2.01 Shiny App. 95% confidence intervals (thick blue dashed line) are displayed around the summary estimate (blue box). The predictive region (thin blue dashed line) shows the 95% confidence area in which the true sensitivity and specificity of future studies lie, taking into account the statistical heterogeneity of the studies shown in this review.

External validation, as a reference for AI implementation in daily clinical practice, was reported in seven of the ten studies (70%), while three of the ten (30%) studies were limited to clinical trials with unclear validation. Figure 3 shows a forest plot of the diagnostic accuracy of all studies. Through bivariate random effects model analysis, the pooled sensitivity of all studies for AI testing was 89.2% (95% CI: 83.7–93.1), and the pooled specificity was 89.7% (95% CI: 81.2–94.6) (Figure 4). Raw confusion matrix data and additional information can be found in the Supplementary Materials File S2.

Differences in reference standards between studies can impact diagnostic accuracy. We also analyzed subgroup outcomes: AI versus PCR and AI versus microscopy. Pooled analysis showed that AI-based malaria detection had higher diagnostic accuracy compared to both PCR and microscopy. When compared to microscopy, AI had a pooled sensitivity of 87.7% (95% CI: 78.2–93.4) and a specificity of 91.4% (95% CI: 77.3–97.1). When comparing AI with PCR as the reference standard, the pooled sensitivity of AI reached 90.7% (95% CI: 83.7–94.9) and a specificity of 88.3% (95% CI: 76.2–94.6). Table 2 illustrates the diagnostic accuracy results for each analysis group. Forest plots for each sub-group analysis are shown in the Supplementary Materials File S3.

## 4. Discussion

This systematic review and meta-analysis involved 6754 patients diagnosed with malaria using AI and gold-standard methods, including microscopic examination and PCR. We assessed the performance of malaria diagnosis using AI compared to gold-standard methods through an external validation approach, which uses an independent dataset to validate the performance of a model trained on initial input data and then tested on significantly different datasets, including different locations, time periods, populations, and dataset sources. This is important because it allows us to determine whether the AI model can be generalized in the real world [27]. Generalizability is the main challenging concept in diagnostic accuracy using AI. The model of AI has been reported with high accuracy during internal validation tests in several studies, but fails to maintain its performance when exposed to external datasets due to domain shift (including patient population, disease prevalence, image features, demographic diversity, scanner hardware, and imaging protocols) [28,29]. Researchers often rely solely on validation to describe internal procedures, such as hyperparameters or compare the performance of models across internal different data subsets to determine the best model [29]. Thus, the external validation approach applied in this study offers significant methodological advantages.

In this study, we incorporated diverse geographic regions to provide an external validation using real-world data. The study encompassed several regions, which were categorized into high-income, non/low-endemic countries (such as United States, United Kingdom, Spain), malaria-endemic countries in Southeast Asia (such as Indonesia, Thailand, Cambodia, Myanmar, Bangladesh), sub-Saharan Africa (such as Ghana, Ethiopia, Nigeria, Kenya, Uganda, and others), Latin America (such as Peru and Brazil), and Oceania (Solomon Islands). Data sources were mostly obtained from travelers, visits from friends and relatives, and migrants arriving from endemic areas. Some data were also collected from hospitals and primary health care facilities in rural and endemic areas. In addition, data sources were also obtained from datasets such as the National Institute of Health (NIH) and the World Health Organization (WHO) External Competence Assessment of Malaria Microscopist (ECAMM) program, which were then tested in different location settings, times, and other dataset sources.

According to the WHO classification for malaria slide readers, which includes four competency levels, with successive parasite detection accuracy: level 1 or expert (90–100%), level 2 or advanced (80–89%), level 3 or competent (70–79%), and level 4 or basic (0–69%), thus ongoing training to improve reader skills [29]. The findings in this study demonstrate that artificial intelligence for malaria diagnosis achieves good diagnostic performance, with overall sensitivity 89.2% and specificity 89.7% when compared with gold standard methods, including microscopic examination and PCR. This shows that the diagnostic accuracy performance with AI is equivalent to WHO level 2 (advanced) expertise. This finding suggests that AI has reached a level of diagnostic accuracy that is clinically meaningful, particularly in malaria-endemic settings when access to expert microscopes or PCR is limited [15]. However, the result of this study also indicates that AI has not yet achieved an expert level based on the WHO classification [30], suggesting both its potential and limitations in clinical implementation.

Based on the results of sub-group analysis, malaria diagnosis using AI compared to microscopic experts or PCR also showed good accuracy, namely 87.7% for sensitivity and 91.4% for specificity compared to microscopic experts and 90.7% for sensitivity and 88.3% for specificity compared to PCR. When converted to the WHO microscopic competence level, malaria diagnosis using AI compared to both gold standard methods is equivalent to level 2 (Advanced), meaning it can be considered at the “reference” level because at this level microscopic experts are expected to provide parasite detection and species identification accuracy of more than 80% and can count the number of parasites within a deviation of 40–25% of the original count (depending on the specific area criteria) [31]. The ability of AI that is equivalent to level 2, rather than level 1 (Expert) can be caused by several things. First, AI is known to be less accurate in determining parasitemia, with parasite quantification often lower than expert microscopist readings [14,20] and even falling short of the WHO’s ±25% true count standard [22]. Second, AI specificity is significantly affected by slide quality; poor staining or artifacts often lead to false positives [23,24]. Furthermore, AI sensitivity decreases significantly in cases with low parasitemia [15,22,25], increasing the risk of false negatives in mild infections. Nearly all studies also emphasize that AI is less able to accurately differentiate *Plasmodium* species, potentially impacting the selection of appropriate antimalarial therapy. In particular, slide artifacts can exacerbate the problem of false positives, decreasing the accuracy of automated diagnosis [23].

Microscopic examination and PCR methods are known to be the gold standard for malaria diagnosis in various regions. Each method has its own advantages and disadvantages. For example, microscopic examination is known to be more economical, facilitates species identification and parasite density through thick and thin smears, and can be stored for long periods. However, this examination requires experienced staff, and its accuracy decreases with low parasite counts [32,33]. PCR is known to be the most sensitive method, capable of detecting even low parasite levels, even below 5 parasites/µL, and accurately identifying parasite species. However, its application is also complex, requires experienced personnel, is more expensive, requires specific reagents, and carries a high risk of cross-contamination [33,34]. A study in India showed that PCR was able to detect 76.5% of positive cases, while microscopic examination only detected 64.4% and rapid diagnostic tests (RDTs) only 63% [11]. Another study in Saudi Arabia also showed that microscopic examination, RDTs, and nested PCR were able to detect positive samples in 10.5%, 12%, and 14.3%, respectively [34]. In this study, AI demonstrated higher diagnostic accuracy compared to PCR. This shows that AI has great potential as a diagnostic support tool that may be comparable to PCR, especially in remote or isolated areas where there are limited microscopy experts or sophisticated PCR equipment.

Beyond diagnostic accuracy, cost–benefit analysis is essential to the implementation of AI-based malaria diagnostics, especially in areas with limited resources. Several clinical fields, such as radiology, pathology, and cardiology, showed that the implementation of AI in clinical diagnosis may require a significant initial investment with annual maintenance expenditures depending on the specialty. However, long-term financial modeling revealed positive Net Present Values (NPVs) in all three specialties, demonstrating that AI-based diagnostic systems consistently improve accuracy and efficiency while generating significant cost savings over time [35]. Although formal cost–benefit analyses for malaria diagnostics are currently unavailable, these findings suggest that utilizing AI for malaria diagnosis could be economically viable, requiring a more prospective economic evaluation.

To our knowledge, this study is the first to assess the diagnostic performance of AI in malaria cases through an external validation approach, meaning the dataset used is independent and reflects performance in a clinical setting. Furthermore, a comparison with two standard methods for malaria diagnosis, microscopy and PCR, provides a comprehensive analysis of AI’s diagnostic accuracy.

## 5. Limitation

This study has various limitations that must be acknowledged. Despite a comprehensive literature search, only ten studies met the inclusion criteria. This small number of studies indicates the scarcity of malaria diagnosis research using AI with an external validation approach. The inclusion criteria, which encompass external validation, are essential for evaluating the clinical significance and generalizability of findings; however, they may impact the precision of pooled estimates. There is also considerable heterogeneity, such as differences in the number of datasets between studies: some studies used only small datasets, whereas others used multicenter field trials with large sample sizes. Furthermore, this meta-analysis is unable to fully meet the three main requirements for malaria diagnosis through microscopic analysis of peripheral blood smears recommended by the WHO, particularly species identification and parasitemia quantification, which are essential for appropriate therapy selection. This study focused solely on the detection of malaria-causing parasites without considering the species and degree of parasitemia. Furthermore, the units of analysis used also differed in each study (slides, patches, pixels, specimens). Therefore, the presence of human analysts is still necessary for final interpretation. One study mentions the concept of human-in-the-loop, where the results of AI algorithms can be corrected by microscopists. This approach has been shown to significantly improve specificity compared to fully automated modes, confirming that at this stage, AI is better positioned as a decision support tool rather than a full replacement for human expertise [17].

## 6. Recommendation

Further research is needed, focusing on low parasitemia conditions, the identification of species and mixed-infections, and employing a multicenter validation in endemic regions to ensure broad clinical generalizability. Furthermore, the involvement of trained human analysts remains indispensable for final diagnostic adjudication and clinical interpretation.

## 7. Conclusions

The use of AI as a malaria diagnostic tool has significant potential to strengthen the malaria diagnostic system. AI is known to have quite good sensitivity and specificity, equivalent to WHO level 2 (advanced), and has diagnostic accuracy comparable to reference standards (microscopy and PCR). However, at this stage, AI is more appropriately positioned as a decision support tool rather than a complete replacement for experts. Therefore, further research and development related to malaria diagnosis using AI to improve diagnostic performance is needed.

## Figures and Tables

**Table 1:** Baseline characteristics of all included studies.

Author, Year and Reference	Location/Setting	Index Test	Study Design	Reference Standard	Data Source	Sample Size	External Validation	Unit of Analysis
Maturana et al., 2025 [15]	Spain	CNN (Yolov5: iMAGING)	Retrospective	Conventional optical microscopy and RT-PCR	Travelers, VFR, and migrant coming from endemic area attending the International Health Unit Drassanes-Vall d’Hebron	46	Unclear	FoV
Liu et al., 2023 [9]	Sierra Leone	CNN (YOLOv5: AIDMAN)	Prospective	Expert microscopist	Prospective: Sierra Leone-China Friendship Hospital. Dataset: NIH Malaria Dataset maintained by the National Library of Medicine	64	Yes	Patches
Nagendra et al., 2024 [14]	United States of America	Deep-Learning (Milab MAL)	Retrospective	Hematopathologist using traditional microscopy	North Carolina, South Carolina, Virginia, the District of Columbia, and Maryland	408	Yes	Pixels
Ewnetu et al., 2024 [20]	Ghana and Ethiopia	Deep-Learning (Milab MAL)	Prospective, Multicenter	q-PCR and expert microscopy	Maraki health center in Gondar, Ethiopia and Agona and Mankranso Government hospitals near Kumasi, Ghana	1650	Yes	Slides
Horning et al., 2021 [21]	Thailand, Kenya, Nigeria, Peru, Indonesia, Cambodia, DR Congo, United Kingdom, United States of America and other countries, Solomon Islands, Myanmar	Easy-Scan Go	Retrospective	Expert microscopy and PCR	WHO External Competence Assessment of Malaria Microscopists (ECAMM) program	55	Yes	Pixels
Rees-Channer et al., 2023 [22]	United Kingdom	CNN (Easy-Scan GO)	Prospective	Expert in manual light microscopy and RT-PCR	Adult travelers, Hospital for Tropical Diseases and Homerton University Hospital, London	1202	Yes	Pixels
Das et al., 2022 [23]	11 countries (Burkina Faso, Kenya, Republic of Congo, Senegal, South Africa, Uganda, Bangladesh, Cambodia, Nepal, Thailand, Brazil)	CNN (Easy-Scan GO)	Multicenter, majority prospective, only South Africa retrospective	Expert microscopy	Endemic area	2250	Unclear	Pixels
Yu et al., 2023 [24]	Sudan	CNN (Malaria Screener) and VF-Net	Prospective	Expert microscopists (WHO Level 1) and n-PCR	Rural hospital, Alsororab and Gezira Slanj, near Khartoum	189	Yes	Pixels
Hamid et al., 2024 [25]	Sudan	Deep-Learning (MiLAB)	Prospective	n-PCR	Primary health care centers at Gezira Slanj (GS) and Alsororab (SOR) in rural Omdurman	190	Yes	FoV
Torres et al., 2018 [26]	Peru	CNN (Autoscope)	Prospective	PCR and manual microscopy	San Juan de Miraflores Health Centre (San Juan), and Santa Clara de Nanay Health Post (Santa Clara)	700	Yes	Unclear

CNN = convolutional neural network; ECAMM = External Competence Assessment of Malaria Microscopists; FoV = field of view; NIH = National Institute of Health; nPCR = nested polymerase chain reaction; PCR = polymerase chain reaction; qPCR = quantitative polymerase chain reaction; RT-PCR = reverse transcription polymerase chain reaction; VF = -net = voxel feature network; VFR = visit friends and relatives; WHO = world health organization; YOLOv5 = you only look once version 5.

**Table 2:** Accuracy of AI diagnosis in malaria.

Group	Total Number of Studies	Sample Size	Pooled Result (95% CI)					Random Effect Correlation
			Sensitivity	Specificity	Diagnostic Odds Ratio	Likelihood Odds Ratio (+ve)	Likelihood Odds Ratio (−ve)	
AI vs. PCR/microscopic examination (overall study)	10	6754	0.892 (0.837–0.931)	0.897 (0.812–0.946)	71.958 (28.744–180.143)	8.637 (4.569–16.326)	0.120 (0.077–0.188)	0.215
AI vs. microscopic examination (sub-group)	9	5273	0.877 (0.782–0.934)	0.914 (0.773–0.971)	75.615 (18.125–315.540)	10.188 (3.539–29.334)	0.135 (0.072–0.252)	0.216
AI vs. PCR (sub-group)	4	3182	0.907 (0.837–0.949)	0.883 (0.762–0.946)	73.259 (22.857–234.801)	7.730 (3.591–16.640)	0.106 (0.057–0.194)	0.225

AI = artificial intelligence; CI = confidence interval; PCR = polymerase chain reaction; vs. = versus.

## Data Availability

The data that support the findings of this study are available from the corresponding author upon reasonable request.

## Supplementary Materials

The following supporting information can be downloaded at: https://www.mdpi.com/article/10.3390/idr18010011/s1, Supplementary Materials File S1: The detailed raw data related to sampling sites, examined species, and comparative diagnostic techniques from each included study. Supplementary Materials File S2: Raw confusion matrix data. Supplementary Materials File S3: Forest plots for sub-group analysis.

## Abbreviations

The following abbreviations are used in this manuscript:

ACT	Artemisinin combination therapy
AI	Artificial intelligence
CI	Confidence interval
CNN	Convolutional neural network
DOI	Digital object identifiers
ECAMM	External competence assessment of malaria microscopists
FP	False positive
FN	False negative
NIH	National institutes of health
nPCR	Nested polymerase chain reaction
PCR	Polymerase chain reaction
PRISMA	Preferred reporting items for systematic reviews and meta-analysis
QUADAS	Quality assessment of diagnostic accuracy
qPCR	Quantitative polymerase chain reaction
RDT	Rapid diagnostic test
RT-PCR	Reverse transcription polymerase chain reaction
SROC	Summary receiver operating characteristic
TN	True negative
TP	True positive
WHO	World Health Organization
