# Supplementary material for: Diagnostic Accuracy of Utilizing Artificial Intelligence for Malaria Diagnostic: A Systematic Review and Meta-Analysis
Source: Infect Dis Rep. 2026 Jan 13;18(1):11. doi: 10.3390/idr18010011 (PMC12821690; doi:10.3390/idr18010011)

Supplementary File 1

The detailed raw data for labelling study according to comparator type (i.e., AI vs. microscopy or AI vs. PCR). The labelling process is important for further sub-group analysis.

Study_ID	Year	Country	AI_Model_Type	Period to follow-up	Dataset_Source	Validation	Comparator_Type (Model vs Expert)		Notes
Das et al	2022	11 countries: Burkina faso, Kenya, Republic of Congo, Senegal, South Africa, Uganda, Bangladesh, Cambodia, Nepal, Thailand, Brazil	EasyScan GO	2018 and 2019	Endemic area	Unclear	Expert microscopist	AI vs Microscopy	-> Normal slide (Microscopy)
Ewnetu 1	2024	Ghana & Ethiopia	Milab MAL	November 1, 2021 and January 31, 2022 (Ethiopia) & January 26 to August 10, 2021 (Ghana)	Ghana and Ethiopia	External	q-PCR and expert microscopy	AI vs PCR	-> Ethiopia (P. falciparum)
Ewnetu 2	2024	Ghana & Ethiopia	Milab MAL	November 1, 2021 and January 31, 2022 (Ethiopia) & January 26 to August 10, 2021 (Ghana)		External	q-PCR and expert microscopy	AI vs PCR	-> Ghana (P. falciparum)

Ewnetu 3	2024	Ghana & Ethiopia	Milab MAL	November 1, 2021 and January 31, 2022 (Ethiopia) & January 26 to August 10, 2021 (Ghana)		External	q-PCR and expert microscopy	AI vs PCR	-> Ethiopia (P.Vivax)
Hamid 1	2024	Sudan	miLab™	October – December 2020	primary health care centers at Gezira Slanj (GS) and Alsororab (SOR) in rural Omdurman	External	nested PCR	AI vs PCR	-> Corrected mode
Hamid 2	2024	Sudan	miLab™	October – December 2020	primary health care centers at Gezira Slanj (GS) and Alsororab (SOR) in rural Omdurman	External	nested PCR	AI vs PCR	-> Automated mode
Horning et al	2021	Thailand, Kenya, Nigeria, Peru, Thailand, Indonesia, Cambodia, DR Congo, United Kingdom, Kenya, USA and other countries, Solomon Islands, Myanmar (detailed locations: Shoklo Malaria Research Unit (Thailand), Amref Health Africa (Kenya), University of	EasyScan GO	N/A	World Health Organization (WHO) External Competence Assessment of Malaria Microscopists (ECAMM) programme	External	Microscopic	AI vs Microscopy	-> EasyScan vs LM

		Lagos (Nigeria), Universidad Peruana Cayetano Heredia (Peru), World Wide Antimalarial Resistance Network (Thailand, Indonesia, Cambodia, and DR Congo), Hospital for Tropical Diseases and the London School of Hygiene and Tropical Medicine (UK), Kenya Medical Research Institute (Kenya), Centers for Disease Control and Prevention (USA and other countries), James Cook University (Solomon Islands), and the Defence Services Medical Academy (Myanmar))							

Liu et al	2023	China	YOLOv5 (AIDMAN)	N/A	Sierra Leone-China Friendhsip Hospital	External	Smartphone smear vs experts	AI vs Microscopy	-> AIDMAN vs Microscopy
Maturana et al	2025	Spain	YOLOv5 CNN (iMAGING)	1 Feb-30 Nov 2023	travelers, Visit Friends and Relatives (VFR), and migrants coming from malaria-endemic areas (mainly Sub-Saharan Africa, South America, and Southeast Asia regions) attending the International Health Unit Drassanes-Vall d'Hebron.	Unclear	Mikrosopi manual + PCR (negative sample)	AI vs Microscopy	-> iMAGING vs LM
Nagendra et al	2024	USA	Milab MAL	Agustus-Sep 2023	North Carolina, South Carolina, Virginia, the District of Columbia, and Maryland	External	Standard microscopy	AI vs Microscopy	-> MiLab vs Microscopy
Rees-Channer 1	2023	UK	EasyScan GO	N/A	Adult travellers, Hospital for Tropical Diseases and Homerton University Hospital, London	External	Expert manual light microscopy and PCR	AI vs PCR	-> EasyScan vs PCR
Rees-Channer 2	2023	UK	EasyScan GO	N/A	Adult travellers, Hospital for Tropical Diseases and Homerton University Hospital, London	Extrenal	Expert manual light microscopy and PCR	AI vs Microscopy	-> EasyScan vs LM

Torres et al 1	2018	San Juan and Santa Clara, Peru	Autoscope	April 2016 to July 2016	San Juan de Miraflores Health Centre (San Juan), and Santa Clara de Nanay Health Post (Santa Clara).	External	PCR	AI vs Microscopy	-> Autoscopy vs microscopy, Santa Clara
Torres et al 2	2018	San Juan and Santa Clara, Peru	Autoscope	April 2016 to July 2016	San Juan de Miraflores Health Centre (San Juan), and Santa Clara de Nanay Health Post (Santa Clara).	External	PCR	AI vs Microscopy	-> Autoscopy vs Microscopy, San Juan
Yu et al 1	2023	Sudan	Malaria Screener (version 1.6.6 smartphone application (Samsung Galaxy A10 + Olympus CX23 microscope).	October 2020 – March 2021	Rural hospital, Alsororab and Gezira Slanj, near Khartoum	External	Expert microscopists (WHO Level 1) and Nested PCR.	AI vs Microscopy	-> Malaria screener vs Microscopy (only in Falciparum and SOR (location)
Yu et al 4	2023	Sudan	Malaria Screener (version 1.6.6 smartphone application (Samsung Galaxy A10 + Olympus CX23 microscope).	October 2020 – March 2021	Rural hospital, Alsororab and Gezira Slanj, near Khartoum	External	Expert microscopists (WHO Level 1) and Nested PCR.	AI vs Microscopy	-> PVF Net (algorithm) ns Microscopy -> post study (whole data)
Yu et al 2	2023	Sudan	Malaria Screener (version 1.6.6 smartphone application (Samsung Galaxy A10 + Olympus CX23 microscope).	October 2020 – March 2021	Rural hospital, Alsororab and Gezira Slanj, near Khartoum	External	Expert microscopists (WHO Level 1) and Nested PCR.	AI vs PCR	-> Malaria screener vs PCR

Yu et al 3	2023	Sudan	Malaria Screener (version 1.6.6 smartphone application (Samsung Galaxy A10 + Olympus CX23 microscope).	October 2020 – March 2021	Rural hospital, Alsororab and Gezira Slanj, near Khartoum	External	Expert microscopists (WHO Level 1) and Nested PCR.	AI vs PCR	-> PVF Net vs PCR

Supplementary File 2.

The confusion matrix of all included studies.
TP = true positive; FN = false negative; FP = false positive; TN = true negative

author	year	TP	FN	FP	TN
Das	2022	846	83	298	925
Ewnetu(1)	2024	145	3	23	141
Ewnetu(2)	2024	169	16	22	567
Ewnetu(3)	2024	65	2	4	160
Hamid(1)	2024	101	11	3	75
Hamid(2)	2024	102	10	26	52
Horning	2021	30	5	0	20
Liu	2023	33	1	0.5	30
Maturana	2025	13	3	3	35
Nagendra	2024	11	0.5	0.5	397
ReesChanner(1)	2023	106	48	114	934
ReesChanner(2)	2023	99	14	122	967
Torres(1)	2018	79	72	75	174
Torres(2)	2018	89	35	27	150
Yu(1)	2023	40	0.5	22	23
Yu(2)	2023	43	5	19	18
Yu(3)	2023	78	18	18	75
Yu(4)	2023	86	13	19	71

Supplementary File 3

Forest plot for sub-group analysis. (1) Diagnostic accuracy between AI versus microscopic examination, which consist of sensitivity (1A) and specificity results (1B), (2) Diagnostic accuracy between AI versus PCR, which consist of sensitivity (2A) and specificity results (2B).

(1) AI vs microscopy

(1A) Forest plot of sensitivity

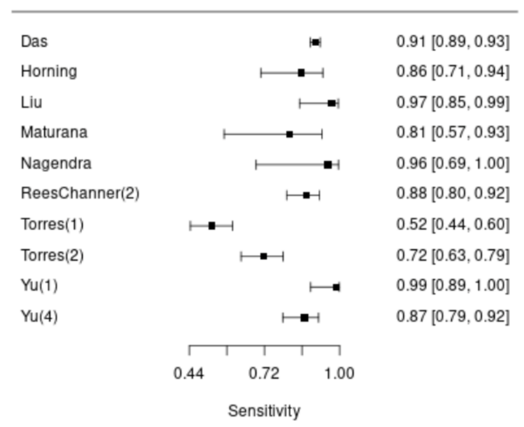

(1B) Forest plot of specificity

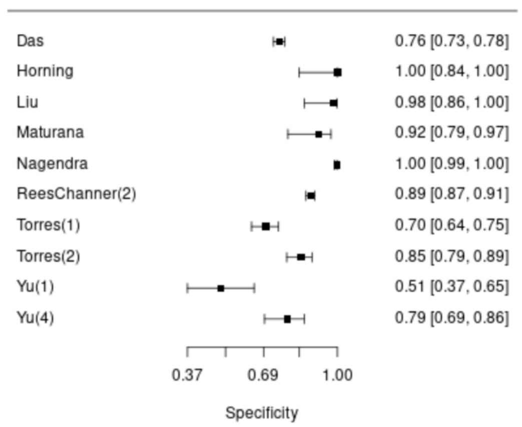

(2) AI vs PCR

(2A) Forest plot of sensitivity

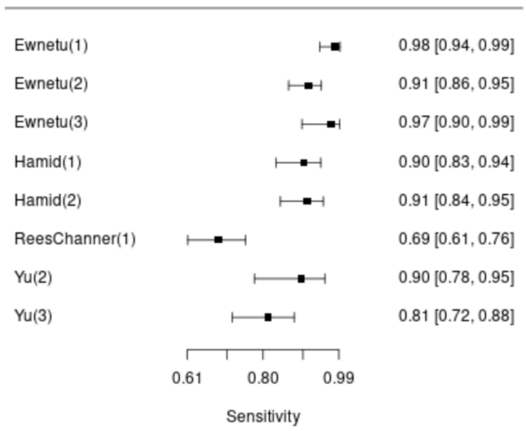

(2B) Forest plot of specificity

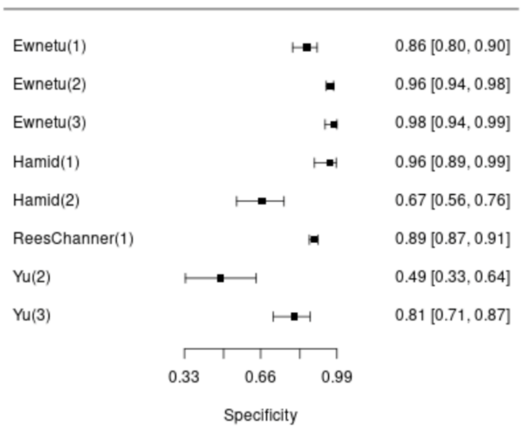

Supplement: Supplementary file 1 [file idr-18-00011-s001.zip › idr-4041928-supplementary.pdf]
